# Cues to cervical cancer screening and reaction to cervical cancer diagnosis during screening among women in Shai Osudoku District, Ghana

**DOI:** 10.3332/ecancer.2022.1392

**Published:** 2022-05-19

**Authors:** Evans Osei Appiah

**Affiliations:** Department of Nursing, School of Nursing and Midwifery, Valley View University, PO Box DT 595, Oyibi, Ghana

**Keywords:** cues, cervical cancer screening, reaction, diagnosis, women

## Abstract

**Introduction:**

Availability and access to cervical cancer screening (CCS) in less developed countries are limited as compared to developed countries. Moreover, the rise in cervical cancer fatalities and mortalities is attributed to the low patronage in CCS among women. The aim of this study was therefore to explore the cues to cervical cancer screening and perceptions of the reaction to cervical cancer diagnosis among women in Shai Osudoku District.

**Methods:**

A qualitative approach and an exploratory descriptive design were considered by the researcher for this study. Seventeen participants in all were recruited to partake in face-to-face in-depth interviews guided by semi-structured interviews. The sampling technique employed is purposive and content analysis was used in the data analysis.

**Findings:**

Two main themes, i.e., cues to cervical cancer screening and perception about women’s reaction to cervical cancer diagnosis, emerged. Health workers, peer influence, spousal influence, creation of awareness and reducing cervical cancer screening cost emerged as major determinants (cues) that influence women’s decision to (not to) screen.

**Conclusion:**

The participants of this study acknowledged that their decision to (not to) screen was greatly influenced by some external factors. Hence, it is recommended that more attention be directed towards motivating and encouraging women to partake in cervical cancer screening services to help reduce fatalities.

## Introduction

Availability and access to cervical cancer screening (CCS) in less developed countries are limited as compared to developed countries [1]. For instance, in Canada, it was discovered most women agree to go for CCS; however, the willingness (or not) to screen may be influenced by a woman’s educational level [2]. The occurrence of cervical cancer in Ghana keeps rising, with higher incidences and deaths recorded in the Greater Accra region of Ghana [38]. A study in Ghana revealed that the perception that women are at risk of cervical cancer serves as a cue for cervical cancer screening [32]. Other authors in Ghana also found that women who have the notion that cervical cancer is serious and CCS is beneficial will be motivated to go for CCS [17]. Spousal influence has also been identified as a factor influencing CCS in Ghana [39].

Furthermore, some researchers in Ghana have ascertained that being married and having a higher educational status are positive determinants for CCS. Moreover, in relation to the disclosure of screening results, the respondents in that study reported that they were not afraid to disclose the results of the screening with their partners and they had a strong conviction that their partners would accept and understand whatever comes out of the test. They further suggested that awareness of CCS among women should be intensified to help women overcome factors that hinder their participation in CCS. Additionally, Tonelli *et al* [40] and Maar *et al* [3] discovered that strengthening women’s knowledge on CCS and organising screening services in the community could increase their participation in screening.

In Kenya and Somalia, the cues to action that influenced women to seek cervical cancer screening uptake were their relatives who died of cervical cancer, mass media and encouragement by health workers or family members to go for screening [4–6]. Similarly, Ackerson *et al* [7] discovered that some cues, such as beginning sexual intercourse at an early age and being in long-term relationships, influenced women to go for routine CCS. Adanu *et al* [8] showed that the cues to action influencing women in Ghana to go for CCS were high educational level, high socioeconomic status and history over the past month of postmenopausal or intermenstrual bleeding. A more recent study in Ghana identified radio campaigns, being referred by a doctor and fear of getting CC as the factors that motivated women to go for CCS [9].

Kuguyo *et al* [10] posited that the burden associated with malignancy of cervical cancer is still on the increase in some African countries, such as Zimbabwe. According to them, the rise in cervical cancer fatalities in Zimbabwe is a result of the prevalence of HIV and inadequate resources for CCS. This is attributed to the decreased patronage in CCS among women aged 20–24 years [11].

Furthermore, researchers have found that cervical cancer screening is not universally available [12], and according to the UNFPA [41], the few institutions where these services are available in Africa are too costly for many women to afford. Shai Osudoku District in Ghana where the study was conducted, as per the Poverty Mapping Report by the Ghana Statistical Service in 2015, is the poorest district in the Greater Accra region. In Ghana, CCS is costly and is not covered by the NHIS; it is expensive for the women to afford, even if they are willing to. Buttressing this, it has been suggested that women who are poor and do not have NHIS are at a greater risk of developing cervical cancer [13]. It is therefore important for health staff to understand the decisions of women in relation to CCS and to enable healthcare providers to guide women towards going for CCS [14, 15]. The researcher therefore aims to explore the cues and perceptions of the reaction to cervical cancer diagnosis among women in Shai Osudoku District

## Research design

A qualitative approach and an exploratory descriptive design were considered by the researcher for this study. A purposive sampling method was selected for this study. It is the process of sampling participants in a qualitative study based on the researcher’s own judgement. It allows the selection of participants who know something about the phenomenon and meet the criteria of inclusion. Hence, this technique allowed the researcher to select participants who were eligible and capable of providing detailed and factual data for analysis. Seventeen participants were selected for this study based on saturation, and so the data collected was repetitive. Saturation ensures that adequate and quality data are collected to support the study [27].

The population consisted of women residing in selected communities in Shai Osudoku District. Women who were willing to participate in the study, were within their reproductive age and could express themselves in English or Twi were recruited. Fortunately, all participants were able to speak and understand English. Excluded from the study were women who were sick during the period of data collection and those with mental health challenges. Shai Osudoku District is located in the south-eastern part of Ghana in the Greater Accra region. The population of the district is projected to be about 67,105, with 32,680 males (48.7%) and 34,425 females (51.3%). The district has 250 communities and the main occupation of the people is agriculture.

The researcher used a semi-structured interview guide to conduct in-depth face-to-face interviews where primary data were obtained from the participants. This was designed by the researcher, reviewed by other researchers and piloted to make sure there were no ambiguities. It was structured into three sections (A, B and C). Section A of the interview guide captured the participants’ demographic data where participants were asked to talk about themselves. The rest of the sections of the interview guide were developed based on the objectives of the study: What influences women to go for CCS? What influences your decision to go for CCS? What will be your reaction upon positive or negative cervical cancer results?

The researcher collected the data himself through face-to-face in-depth interviews, which were audio recorded. The data collection commenced following ethical clearance granted by the Noguchi Memorial Institute for Medical Research Institutional Review Board (NMIMR-IRB) of the University of Ghana with protocol number NMIMR-IRB 036/18-19. After ethical clearance, permission was sought from the District Chief Executive before entering the community. Participants were selected from various gatherings and functions (churches and marketplaces) that took place within the selected communities. Permission was first sought from the leaders of the churches and the market secretary before contacting the market women and the congregants. Participants were informed about the exercise and the purpose of the study. Both verbal and written consent were obtained prior. Contact numbers of those who were willing to participate in the study were collected to arrange for a place and time of meeting. The researcher visited the clients at the agreed time and private place to conduct the interviews. The interviews lasted for 30–45 minutes each. Data collection lasted for a month.

The trustworthiness (methodological rigor) of this study was maintained by adhering to the following principles: credibility, dependability, transferability and confirmability. These were ensured by pre-testing the interview guide with four women from the Oyibi community in order to revise and make additions where appropriate. Moreover, an expert in English language and colleagues were allowed to review it to shape the work to make it more scholarly. Feedback from the Institutional Review Board was factored in and corrections were sent back as required to ensure quality work is produced at the end. Recorded data were also transcribed verbatim.

**Figure 1.**
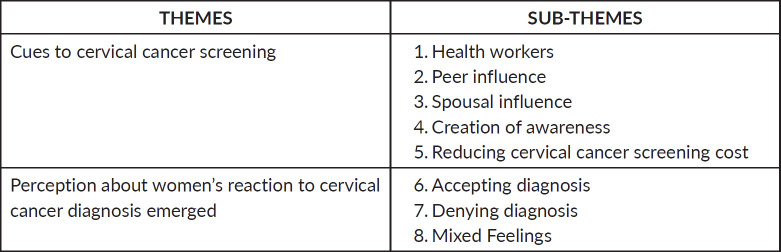
Themes and subthemes.

The data was analysed by the researcher using content analysis. After it was reviewed by other colleague researchers, they provided effective input. This was carried out through the process of familiarisation, condensation, categorisation, coding the text and grouping codes into themes and subthemes. With this, the researcher first transcribed all the 15 interviews into a word document, then read through the transcripts repeatedly to gain a detailed understanding of the transcripts, The text of the transcripts was then shortened by two to four words while preserving the meaning of the participants. These words were categorised by grouping the ones with similar meaning together, and they were finally grouped into themes and subthemes. In all, two main themes and eight sub-themes were generated, as shown in Figure 1.

## Results

In this section, the participants’ demographic data have been highlighted, as well as the description of the themes and sub-themes that emerged.

### Socio-demographic characteristics of participants

In total, 17 participants took part in this study. The participants were within the age bracket of 22–45 years. More than half of the participants (*n* = 11) were below the age of 30 years. The women who were married were more than the spinsters in this study (10 and 7, respectively). Almost all the participants (15) had some formal education. The other details are presented in Table 1.

### Organisation of the themes and sub-themes

The data analysis revealed two main themes and eight subthemes. The two themes were cues to cervical cancer screening and perceptions about women’s reaction to CC diagnosis. The two themes and their emerging sub-themes are shown in Figure 1.

### Theme 1: Cues to cervical cancer screening

Cues refer to factors that motivate or influence women to participate in cervical cancer screening. Participants in this study listed some cues that influence women either negatively or positively in seeking cervical cancer screening. The cues mentioned by participants were health workers, peer influence, marital status and the media.

### Health workers

Most participants believed that health workers play a significant role in increasing women’s participation in cervical cancer screening. This is evident in the statements made by the following women:

*If health workers from various health facilities could put it upon themselves to at least go round once a month to educate the community on CC and CCS just as they have done for polio and other diseases, it would draw their attention on it and more women would go for it.* **(Akua, 24 years)**

*Once your health professionals tell women about the possible death that could occur, so you tell us it could end our life…there will be awareness, there will be…what should I call it? A need for women to go and check-up to see if they are having this or not.* **(Abena, 25 years)**

Rosina was of the view that the attitude of health workers could be either a positive or a negative influence on CCS. This is how she narrated it:

*I think how health professionals relate to we patients can either encourage more woman to go or not to go for the screening, because sometimes when you just go to some of our hospitals that you are not sick but you are just coming for screening, how they will treat you, you might never want to go to the hospital for screening again.* **(Rosina. 34 years)**

A 25-year-old participant was of the view that, apart from doctors educating more women to go for the screening, they themselves should be educated on it so that they can influence more women to go for the screening. This is what she has to say:

*When we go to the hospital for treatment for other sicknesses, the doctors there should educate the women about CCS as well as tell them something little about it so that we will be aware of what it is and go for it. It is also necessary for the health professionals themselves to be educated about it so they can know about it and educate other women.* **(Adwoa, 25 years)**

### Peer influence

Apart from health professionals, peer influence was another factor indicated by participants to positively influence women to seek cervical cancer screening services, even though some had a contrary view that friends could discourage women from participating in the cervical cancer screening. The following statements were expressed by some of the women:

*I think friends can influence their peers who are women to go for it. You know how women are, the way they can gossip, if they hear of something how they will say it, so even if their colleagues have not decided, they can make them decide by convincing them.* **(Afia, 26 years)**

*Friends, you see errm people we are closer to, have a lot of influence in our lives, maybe you have a friend whom you trust so much so whatever the person tells you, you know if this your friend do it and come to tell you about it that is not anything serious you will also want to go and do it just because your friend has done it.* **(Abena, 25 years)**

Some participants expressed that they will be encouraged to partake in the screening if their close friends suffered from CC.

*Ok, as you know…as in we are all here and then I hear that this my friend is suffering from this sickness. So she went to the hospital and then they got to know that she had cervical cancer. I will say eiiii! I have not done it before so let me try it, let me try*. **(Yaa, 28 years)**

Few participants shared a contrary view that sometimes friends can discourage their colleagues from going for the screening. This is illustrated in the following statement:

*Sometimes, Friends can discourage you from coming for the screening as in…… maybe you are thinking of going and the kind of things that your friends will start saying like, if you go and you get it you will die and you can’t also continue chilling again and they will be scaring you. This can prevent women from going for the screening.* **(Rose, 28 years)**

### Spousal influence

While the majority of women believed husbands could be of good influence to their wives in seeking CCS, others thought that husbands will prevent their wives from partaking in the screening due to some reasons listed. This is evident in the following statements:

*I think those who are married their husbands can encourage them, to go for this screening, most especially men who are well enlightened and knows about this cancer.* **(Akosua, 30 years)**

*I think all men should encourage their wives to go for the screening. If you love your wife you should be able to allow her go for the screening so that she will have good health. Also, whether you are married or not, we should all go.* **(Rafiatu, 45 years)**

A 29-year-old woman had mixed feelings about these cues to CCS.

*Some men are jealous and will not allow other men to look at their wives’ private parts whilst other men who are educated and care so much about their wives health will not mind their wives going for it. But sometimes if you are not married like me no one controls me so I decide whether to go for it or not to go for it.* **(Ramatu, 29 years)**

### Creation of awareness

Increasing the knowledge on CCS/CC was one of the strategies suggested by participants to increase cervical cancer patronage in Ghana since they believed most Ghanaian women are unaware of the screening. Some women suggested that there should be increase education on cervical cancer screening in the media.

*I think it is because most women don’t have knowledge about it, so errm, so publicising it on TV and Radio, I think, will reduce these barriers. They should educate women too, the young women in schools and tertiary institution, to help them to be aware of the screening. Creating awareness on the media thus TV, radio etc. especially on special occasions such as mother’s day etc. can help more women to be aware so that they will willingly go for the screening.* **(Adwoa, 25 years)**

*It is not about education on TV alone but they should show us how the screening is done to clear the misconceptions that some of us have so that we all can come for the screening and sometimes too what we read on the internet have so much influence on us to or not to engage in the screening.* **(Ruth, 37 years)**

Other women suggested that flyers could be created as a means of strengthening education on CCS/CC.

*I think creating awareness of it, doing advertisement and all that, some people ones they see an advertisement, they know that this is very beneficial for their kids so they will allow their kids to get involved in it. And also, spreading the news about the screening as used in marketing as in creating flyers on the screening*. **(Ama, 29 years)**

30-year-old Akosua proposed that educating women in their various local languages can help increase screening patronage.

*We should give more education like, TV should do their work, the media, and people should, we should educate women and translate it to different languages so that people will be aware, … if they understand what is the meaning … and you ask them to come for the screening they will go.* **(Akosua, 30 years)**

Few of the participants shared that educating men also can reduce the obstacles to CCS.

*I will say awareness campaigns, and then you educate both the old and the young, and even males about this kind of disease because maybe a female might be having it and the possible signs may show but will not know and a male who knows about it might be able to give a helping hand and encourage them to seek treatment.* **(Abena, 25 years)**

*Reducing cervical cancer screening cost*

Some women had the perception that if cervical cancer screening cost is reduced, more women will patronise cervical cancer screening, especially those who are poor. This is depicted in the expressions of some participants.

*I think the government should also make policies to subsidise the screening cost, the drugs, and other treatment interventions by making it easy and accessible so that everyone can afford if possible, it should even be made free so that more women can go for this screening*. **(Adwoa, 25 years)**

Other participants shared that the screening cost should be covered by the NHIS.

*I think with the NHIS more women will go for the screening because sometimes some Ghanaians actually go to the hospital without any money just the insurance, so if the insurance covers the screening, I guess more women will go for it*. **(Ama, 29 years)**

*If it covers it is good because we Ghanaians mostly like free things or cheaper or less expensive things, so if the NHIS insurance covers more women will go for it.* **(Rita, 22 years).**

A few suggested that husbands should pay for their wives to motivate them to go for the screening.

*If you a man and you have a wife you should either pay for your wife or support your wife to pay for the screening so that they will be motivated to go for it and for them not to transfer it to you if they have it.* **(Akua, 24 years)**

Participants indicated that for the willingness to go or not to go for CCS is highly influenced by one’s peers, marital status, finances, level of awareness on CCS and motivation from health workers

### Theme 2: Perception about women’s reaction to CC diagnosis

Reaction to cervical cancer screening was a major theme that emerged based on the responses from participants outside the constructs of the theory. This section deals with the perceptions of how women react to being diagnosed with cervical cancer. Participants in this study expressed how they will behave or what they will do in case they are diagnosed with cervical cancer. Their responses were grouped into whether they would accept their diagnosis and move on, deny their diagnosis or demonstrate mixed feeling.

### Accepting CC diagnosis

Most of the participants had the perception that they will accept positive cervical cancer screening results and move on with their life by going for treatment. The reasons mentioned by the participants with regard to why they will accept the results are as follows: they cannot change the results because there is treatment available for CC; because they do not have a choice; and have a belief that everyone will die. This is demonstrated in the following expressions:

*I will just go cool with it and ask for some help because I know the doctors can help me so I will ask them for how possible they can help me because you know that once you are told you have it, no matter what you do it won’t change the situation so I just have to take it like that and look for a way out****. ***(**Akua, 24 years)**

*I wouldn’t really be much worried about it because I have been told, is different from not visiting the hospital at all and then waiting for say 4 or 5 months and then later when the thing is severe, you are told you have it and it has destroyed this organ so if I am told I will look for ways of solving it, I will, try and get some medications.* **(Abena, 25 years)**

Some women had the perception that after all every woman will die and hence will accept CC diagnosis, as portrayed in the statement below:

*Hmmm, it is not easy but I will accept. After all, I am 42 years now and I know everybody will die so I will just go and gather money for treatment till I die because whatever I do, I can’t get the condition out of my system.* **(Rejoice, 42 years)**

Rafiatu added that she will accept the diagnosis because it can be cured.

*I have grown to believe that anything can happen in this world at any time, the young, the old can die so if I am diagnosed, I will accept the diagnosis since it can be cured and I will take my medicines to cure it.* **(Rafiatu, 45 years)**

### Denying CC diagnosis

Most people do not initially want to accept the diagnosis of a serious or life-threatening illness. It was therefore not surprising that some participants in the current study reported that they will refuse the diagnosis of cervical cancer and never wished to be diagnosed of it in their lives with the reason that it is deadly and has no cure. Others shared that they will reject positive CCS results on the basis that they are too young to contract CC and diagnosis of it means that one is cursed. This is illustrated in the following narrations:

*God forbid, I will have collapsed (laughing). Because I am too young to get this kind of cancer, cervical cancer so I won’t accept it and no one in her right senses will accept this kind of disease and I am not planning to die anytime soon so I reject this kind of disease.* **(Ramatu, 29 years)**

*I won’t accept this because accepting it will make you feel sad because all of a sudden you are diagnosed with this cancer. Like hmm, it will be very difficult for me to believe.* **(Afia, 26 years)**

A 34-year-old participant likened cervical cancer diagnosis to an HIV diagnosis and declared that she will not accept the diagnosis.

*When I am told I am sick of cervical cancer, of course, I will not accept it. Because the diagnosis of cervical cancer is just like HIV. You will feel sad; you will feel down, you will feel like the world has ended so for me I will not accept it.* **(Rosina. 34 years).**

Ama shared that she will rather commit suicide than to accept cervical cancer diagnosis.

*If I am told I have CC I think I will be surprised, And I will be like why?...how? What went wrong and I will be in shock and I will rather commit suicide than to live with it.* **(Ama, 29 years)**

### Mixed feelings

Some participants had mixed feelings concerning their reaction to cervical cancer diagnosis by saying that even though it will be difficult for them to accept it initially, as time goes by they will learn to accept it and move on. The following are the expressions to show mixed feelings as shared by some participants:

*Ha-ha, ok, first I will be in a state of shock, as in I will ask questions like, how could I get it? So, at first, I won’t accept it, I will try and go to other hospitals to find out but as time goes on, I will learn to accept it and then find the treatment to it that is if really there is the treatment* **. (Adwoa, 25 years)**

*Hmm, I will not believe it because I am too young to suffer this kind of disease. But if it is really true, I will keep it to myself and wait for me to die because I learnt it has no cure, right? And also, if you tell people they are going to gossip about you and spread the news so I will keep it to myself.* **(Yaa, 28 years)**

32-year-old Rexy, who had a perspective that she does not deserve the diagnosis of cervical cancer, indicated that it is better to accept CC diagnosis and treat it early.

*Eiish, cervical cancer! it will be difficult to believe because I have not done anything to deserve that but if it is true that I have it then I think the earlier I start treatment, the better so that it doesn’t become worse.* **(Rexy, 32 years)**

Women in this study also shared their views on how they will react if their screening comes out positive. Majority indicated that they will reject the results, while some made it known that they have no option but accept the results. Others also have mixed feelings.

## Discussion

### Cues to cervical cancer screening

Cues are factors that encourage women to go for CCS. Almost all participants in the present study believed that the rate at which women participate in cervical cancer screening could be influenced by factors including the media, friends, family members and health workers. Similar to this finding is a study by Ebu and Ogah [17] in Ghana, who identified that there are some cues that influence women’s participation in CCS and added that the higher the cues, the higher the probability to partake in CCS. This implies that more attention should be redirected to these factors in order to increase CCS utilisation.

A health worker was a significant cue that was identified by the majority of participants in this study to increase the willingness of women to engage in CCS. According to participants, health workers play a vital role in the creation of awareness on CC/CSS, thereby increasing CCS uptake. Other women in the present study were of the view that a positive relationship between health professionals and their patients can motivate more women to partake in CCS. This finding is congruent with several other studies supporting the idea that physicians play a significant role in increasing CCS uptake [18, 19]. For instance, encouragement received from health workers was detected to motivate women to partake in CCS [20]. Although the present study is not directly linked to a study by Bukirwa *et al* [42], it has some relationship since participants in this study thought that some women are motivated to seek CCS with the notion that other physical exams will be carried out by health workers during the process of the screening. Consistent with the findings of the current study is a study by Morema *et al* [21], whose results showed that the majority of women who send their children to the Child Welfare Clinic are motivated more to participate in CCS than those who are not. This implies that health workers in Ghana and worldwide should do their part by educating more women on CC and CCS in order to improve CCS uptake in Ghana. Moreover, health workers should maintain a positive nurse–patient relationship with all women reporting to the hospital for CCS since it will help increase CCS utilisation in the long term.

Furthermore, peer influence was identified as another factor encouraging more women to seek CCS services. Friends have a major influence on the various decisions and choices of their peers, including health. Hence, the majority of participants in the current study indicated that friends have a positive influence on women’s willingness to seek CCS. Participants based their argument on the fact that friends will gossip or make noise about the screening when they hear about it to other friends to make more women aware and go for it. Other participants reported that some women are motivated to go for the screening by the mere fact that their friends have gone for it or have been diagnosed with CC. In this finding, friends were identified as being influential in CCS uptake [22]. For instance, Matejic *et al* [23] reported that women who engaged in conversation with other women who had cervical cancer were more likely to go for cervical cancer screening. On the contrary, Moore de Peralta *et al* [18] identified mothers (63%) as having a higher influence on their daughters’ willingness to participate in CCS.

It was discovered that spousal influence is a cue to CCS. The majority of participants in the current study suggested that husbands can influence their wives positively to seek CCS. They added that husbands who are more enlightened and love and understand their wives more will influence their wives more than those who are not. Comparable to findings of the current study by Ncube *et al* [24] in Jamaica, Visanuyothin *et al* [25] unravelled that being married is a positive determinant to CCS uptake. For example, husbands were identified, by Kim *et al* [22] and Adegboyega *et al* [26], as influencing their wives to participate in cervical cancer screening as well as cervical cancer treatment. In contrast to the present study, a study by Widiasih and Nelson [28], although reporting that Muslim husbands support their wife’s health in various aspects, with respect to cervical cancer screening, the participants indicated that there were limited support and influence from their husbands. Husbands are therefore to be educated and encouraged at various gatherings on the need to support their wives to partake in CCS to help reduce the incidence of CC in Ghana.

Finally, the media was suggested by most participants in this study as a key motivator for CCS uptake. The media types listed to influence women to partake in CCS were TV and radio. The media as a cue to cervical cancer screening as indicated by participants in the present study was supported by Peralta *et al* [18]. The findings of the current study were supported by Teng *et al* [29], which ascertained that media campaigns can help overcome impediments to CCS, such as shyness and increase in CCS uptake. It was therefore proposed by Ziba *et al* [30] that there was a need to strengthen education on CC/CCS in the media to increase utilisation of CCS.

An equally important finding of this study is the fact that participants identified that reducing CCS charges could increase CCS patronage. Others suggested that the government should not only help to subsidise the screening cost but also the cost of treating cervical cancer. Other women in this study recommended the need for the CCS cost to be rolled onto the NHIS. This is logical because people always want to patronise services that are cheap. Furthermore, Ghana is considered a middle-income country and this study were conducted among women living in rural communities in Ghana who are considered poor, so this suggestion by participants was not out of order. This finding is not surprising since several studies have identified high charges as an impediment to CCS uptake [41, pp 31–33]. The findings of this study agree with the findings of Chang *et al* [14], who found that how rich or poor a person is can have an influence on their willingness to engage in cervical cancer screening. For example, *et al* [30], in Ghana, suggested that more women will go for CCS if the cost is covered by NHIS. This is true because some Ghanaians come to the hospital with only their NHIS to seek care.

### Perception about women’s reaction to CC diagnosis

Being diagnosed with a life-threatening disease is one of the most difficult life experiences patients encounter and find it difficult to cope with. Participants in this study expressed various reactions to cervical cancer screening diagnosis which were classified as accepting the diagnosis, rejecting the diagnosis or having mixed feelings.

Even though being diagnosed with cancer may cause psychological trauma and anxiety to women affected, some participants in the present study reported that they will accept to go for cervical cancer screening and accept the results even when it is positive. They added that after accepting the results, they will find ways of dealing with it so that their condition does not worsen. Similarly, Patricia *et al* [34] found that cervical cancer screening diagnosis causes distress to individuals affected but recommended the need for them to accept the diagnosis and adopt ways of dealing with it properly. Relatedly, Rosser *et al* [35] revealed that some men, even though they disclosed that they will go through emotional trauma upon hearing that their partners are diagnosed with cervical cancer, they were still willing to encourage their partners to go for the screening. Women who are aware of CCS and CC are more likely to accept CCS uptake [36]. For the women who professed that they accept CC diagnosis, their reasons are probably due to the fact that they are enlightened about it and believe in orthodox interventions.

Some participants in the current study ascertained that they will reject a positive cervical cancer screening results due to the emotional stress associated with it to the extent that some preferred committing suicide to living with cervical cancer diagnosis. Similar findings of the present study are disclosed by Binka *et al* [43] in that individuals diagnosed with cancer try to cope with the stress associated with it by rejecting the diagnosis. On the contrary, Viviano *et al* (2017) found that some of their participants who were diagnosed as HPV positive through CCS and referred to undergo colposcopy accepted and went, while quite a few refused to go for the screening. The findings of a study by Kivuti-Bitok *et al* [37], even though not directly related to the present study, revealed that some physicians even have difficulty disclosing positive cervical cancer screening results for the fear of causing emotional stress to their patients. Rejection of CC diagnosis by these participants could be attributed to the fact that they have poor knowledge regarding CC/CCS and due to some religious and cultural beliefs.

Another cardinal finding related to the perception of women’s reaction to CC diagnosis was that some women in the present study had mixed feelings. They asserted that they will initially reject a CC diagnosis to seek further opinions from different hospitals but will accept later when they cannot do anything about it. This makes sense since nobody initially wants to accept situations that will cause distress or a major change in their lives. This explains why most Ghanaian women wait until they are down with a condition before visiting the hospital.

### Limitations

The study was limited to one district in Ghana and hence future studies should consider women from several districts across the country.

## Conclusion

The participants of this study acknowledged that their decision to (not to) screen is greatly influenced by some external factors. Hence, it is recommended that more attention be directed towards motivating and encouraging women to partake in cervical cancer screening services to help reduce fatalities. Women also fear the diagnosis of cervical cancer, which implies that measures put in place to reduce cervical cancer risk, such as cervical cancer screening, will be embraced by these women. Men should be encouraged to provide support to their partners in terms of motivating them and supporting them financially for the screening to help increase CCS uptake. Further studies are needed on the reactions of women to cervical cancer diagnosis, especially among women diagnosed with cervical cancer.

## List of abbreviations

CC- cervical cancer, CCS- cervical cancer screening, GSS- Ghana Statistical Service, GAR- Greater Accra region, HIV- human immunodeficiency virus, NHIS-National Health Insurance Scheme, SOD- Shai Osudoku District, NMIMR-IRB- Noguchi Memorial Institute for Medical Research Institutional Review Board, UNFPA- United Nations Population Fund

## Conflicts of interest

The author declares that there is no competing interest in relation to the publication of this paper.

## Funding

The authors did not receive any funding for this study.

## Figures and Tables

**Table 1.** Socio-demographic characteristics.

Variable	Frequency (*N* = 17)	Percentage (%)
**Total**	17	100
**Age**		
22–30	11	64.70%
31–35	4	23.50 %
36–45	2	11.80%
**Religion**		
Christianity	16	94.10%
Muslim	1	5.90%
Others	0	0
**Marital Status**		
Single	7	41.20%
Married	10	58.80%
Cohabitation	0	0
Divorced	0	0
**Ethnic Group**		
Akan	10	58.8%
Ewe	2	11.7%
Fante	1	5.9%
Ga	4	23.5%
Others	0	0
**Occupation**		
Students	8	47.1%
Government workers	4	23.5%
Housewives	3	17.6%
Self-employed	2	11.7%
